# Effectiveness of Losartan-Loaded Hyaluronic Acid (HA) Micelles for the Reduction of Advanced Hepatic Fibrosis in C3H/HeN Mice Model

**DOI:** 10.1371/journal.pone.0145512

**Published:** 2015-12-29

**Authors:** Reju George Thomas, Myeong Ju Moon, Jo Heon Kim, Jae Hyuk Lee, Yong Yeon Jeong

**Affiliations:** 1 Department of Radiology, Chonnam National University Hwasun Hospital, Chonnam National University Medical School, Gwangju, Korea; 2 DKC Corporation (BioActs), Incheon, South Korea; 3 Department of Pathology, Chonnam National University Hwasun Hospital, Chonnam National University Medical School, Gwangju, Korea; University of Helsinki, FINLAND

## Abstract

Advanced hepatic fibrosis therapy using drug-delivering nanoparticles is a relatively unexplored area. Angiotensin type 1 (AT1) receptor blockers such as losartan can be delivered to hepatic stellate cells (HSC), blocking their activation and thereby reducing fibrosis progression in the liver. In our study, we analyzed the possibility of utilizing drug-loaded vehicles such as hyaluronic acid (HA) micelles carrying losartan to attenuate HSC activation. Losartan, which exhibits inherent lipophilicity, was loaded into the hydrophobic core of HA micelles with a 19.5% drug loading efficiency. An advanced liver fibrosis model was developed using C3H/HeN mice subjected to 20 weeks of prolonged TAA/ethanol weight-adapted treatment. The cytocompatibility and cell uptake profile of losartan-HA micelles were studied in murine fibroblast cells (NIH3T3), human hepatic stellate cells (hHSC) and FL83B cells (hepatocyte cell line). The ability of these nanoparticles to attenuate HSC activation was studied in activated HSC cells based on alpha smooth muscle actin (α-sma) expression. Mice treated with oral losartan or losartan-HA micelles were analyzed for serum enzyme levels (ALT/AST, CK and LDH) and collagen deposition (hydroxyproline levels) in the liver. The accumulation of HA micelles was observed in fibrotic livers, which suggests increased delivery of losartan compared to normal livers and specific uptake by HSC. Active reduction of α-sma was observed in hHSC and the liver sections of losartan-HA micelle-treated mice. The serum enzyme levels and collagen deposition of losartan-HA micelle-treated mice was reduced significantly compared to the oral losartan group. Losartan-HA micelles demonstrated significant attenuation of hepatic fibrosis via an HSC-targeting mechanism in our in vitro and in vivo studies. These nanoparticles can be considered as an alternative therapy for liver fibrosis.

## Introduction

Hepatic fibrosis is an illness affecting a large number of people, and it asymptomatically leads to cirrhosis primarily due to chronic hepatitis virus infection, alcohol abuse, and non-alcoholic fatty liver diseases, and causes 30,000 deaths in USA alone [1]. Further injury to the liver leads to cirrhosis, in which the architecture of the functional units of the liver becomes disrupted, leading to complications such as portal hypertension or even hepatic cellular carcinoma (HCC). HCC associated with cirrhosis is now considered to be among the top ten causes of death worldwide [2, 3].

The renin-angiotensin system (RAS) is well-known to play an important role in hepatic fibrosis. RAS components are overexpressed in hepatic fibrosis, one of which is angiotensin II overexpression that give rise to fibrogenic and inflammatory effects in activated hepatic stellate cells (HSC) and *in vivo* through angiotensin type 1 (AT1) receptors [4]. In the normal human liver, HSC do not express AT1 receptors, nor do they secrete angiotensin II. Therapies which involve the strategy of targeting the RAS can be modelled for hepatic fibrosis treatment [5, 6]. Thus, the AT1 receptors blockade can reduce the activated HSC accumulation and attenuates liver fibrosis in rats [7].

Though various therapeutic strategies have been applied to the reversal of hepatic fibrosis, no drug that fulfills this purpose has yet been successfully introduced [8, 9]. Losartan is an angiotensin II receptor blocker that acts upon AT1 receptors [10] and is coupled to HSC-specific carriers [11, 12]. Losartan has been found to inhibit the progression of hepatic fibrosis [13]. The compound is a major candidate in clinical studies as an antihepatic fibrosis drug [6].

The present form of losartan is lipophilic and therefore cannot be administered intravenously for improved bioavailability [14]. Losartan was conjugated to mannose 6-phosphate-modified human serum albumin (M6PHSA) via a platinum linker, which was administered to a CCl_4_-treated rat model for hepatic fibrosis. Losartan-M6PHSA reduced advanced hepatic fibrosis in a short term study [12]. Linker-conjugated losartan therapeutics can deliver only a small amount of drug to the target site compared to micellar systems.

Hyaluronic acid (HA) is a glycosaminoglycan that is abundantly found in animal extracellular matrix, connective tissue and organs [15]. HA is a biocompatible, biodegradable, nonimmunogenic and noninflammatory, nontoxic, and linear polysaccharide [16]. CD44 expression is also increased in cases of hepatic fibrosis [17, 18]. CD44 have an important role in activated HSCs migration during liver injury [19]. CD44 is a suitable target for HA receptor-mediated drug delivery systems [20]. For this specific purpose, we have selected a HA polymer backbone to develop micelles and deliver losartan via a CD44 receptor-based targeting mechanism.

A previous study reported the feasibility of HA derivatives as novel drug delivery carriers for the treatment of various chronic liver diseases, including hepatitis and liver cirrhosis [21]. Here, the main objective of the study was to evaluate the effect of losartan-loaded HA micelles as a targeted therapy for hepatic fibrosis in a mouse model. To verify the effect of losartan-loaded HA micelles, we evaluated the effects of a short-duration micelle treatment in a C3H/HeN mouse hepatic fibrosis model.

## Materials and Methods

### Materials

Sodium hyaluronate (0.48 MDa) was purchased from Bioland, Korea. 5ß-cholanic acid (CA), Formamide and Pyrene was purchased from Sigma Aldrich, USA. Fluorescent probe Flamma^™^FCI-774 (F774) and Flamma^™^FCR-552 (F552) were obtained from BioActs, Korea. 1-Ethyl-3-(3-dimethylaminopropyl) carbodiimide (EDC), N-hydroxysuccinimide (NHS) and dicyclohexylcarbodiimide (DCC) were purchased from Sigma Aldrich, USA. N-N dimethyl formamide was purchased from Merck, Germany. 3-(4, 5-dimethylthiazol-2-yl)-5-(3-carboxymethoxyphenyl)-2-(4-sulfophenyl)-2H-tetrazolium) (MTS) was purchased from Promega, USA. Losartan potassium(Sigma Aldrich, USA), Angiotensin 2 human Sigma Aldrich, USA), Anti alpha smooth muscle actin antibody (abcam,Cambridge,UK),Donkey Anti-Rabbit IgG H&L (Alexa Fluor^®^ 488) (abcam,Cambridge,UK), Goat anti-rabbit IgG (HRP) (abcam,Cambridge,UK), DAB chromogen (Dako, Agilent Technologies, Denmark). FL83B cell line was purchased from ATCC (Manassas,USA) and hHSC from ScienCell Research Laboratories (CA,USA). Hydroxyproline Assay kit (Chondrex,WA,USA). RPMI-1640 and Dulbecco’s modified Eagle’s medium (DMEM) were purchased from Thermo Scientific, USA. All other reagents were of analytical or chromatographic grade.

### Synthesis of Losartan-Loaded HA (Losartan-HA) Micelles

We synthesized the HA conjugated to 5ß-cholanic acid (CA), as described elsewhere [22]. Briefly, 500 mg of 5ß-CA was dissolved in 5 ml of methanol. Then, 1 ml of 37% HCl was added and refluxed at 60°C for 6 hours. The mixture was cooled to 0°C to obtain a white precipitate, which was filtered out using a membrane filter (pore size: 0.45 μm, Millipore). The Filtrate was vacuum-dried and was dissolved in 5 ml of ethylene diamine (EDA) and refluxed at 130°C for 6 hours. The mixture was cooled to room temperature to obtain aminoethyl 5ß-cholanomide (EtCA) as a white precipitate. Next, 120 mg of HA (0.48 MDa) was dissolved in 70 ml of formamide by overnight stirring, and 48.5 mg of EDC (*N*-(3-Dimethylaminopropyl)-*N*′-ethylcarbodiimide hydrochloride) was then added and stirred for one hour, after which 29.1 mg of NHS was added. Subsequently, 0.26 mg of EtCA dissolved in 28 ml of dimethyl formamide was added dropwise to the HA solution and stirred for one day. The resultant product was dialyzed against a water/methanol mixture for 2 days using a cellulose ester dialysis membrane bag (Spectrum lab, CA,USA) of MWCO = 3500 to remove unreacted chemicals, followed by 2 additional days of dialysis against water alone. To obtain the micelles in a powder form, the sample was cooled in liquid nitrogen and lyophilized at 0.01 mBar and -81°C for 5 days (Labconco FreeZone, Kansas, USA).

To prepare losartan-HA micelles, we utilized an oil-in-water emulsion method by starting with 10 mg of HA micelles dissolved in distilled water. One milligram or three milligrams of losartan was dissolved in 100 μl of 100% ethanol and was added drop-wise to the HA micelles in water and stirred for 24 hours. Next, the losartan-HA micellar solution was centrifuged at 5,000 rpm for 10 minutes. The supernatant was discarded, and the obtained pellet was dissolved in acetonitrile (200 μl) and probe-sonicated for 10 minutes at a 2:3 pulse rate to disrupt the micellar structure. Losartan that was freely soluble in acetonitrile was analyzed by HPLC using an acetonitrile mobile phase (20:80, v/v). The encapsulation efficiency was calculated as the ratio of the amount of losartan contained within the micelles to the total amount of losartan added in the solution, and the drug loading capacity was determined based on the total amount of losartan in a specific amount of micelles [23]

### Characterization of Losartan-HA Micelles

The lyophilized HA micelles were subjected to ^1^H-NMR analysis to study conjugation level between the HA and EtCA. To characterize the morphology of the micelles, hydrophobic oleic acid-coated superparamagnetic iron oxide nanoparticles (SPIONs) were loaded into the HA micelles, at 10:3 (HA micelle:SPION) ratio and then visualized by transmission electron microscopy (TEM). Scanning electron microscopy (SEM) images were taken using Hitachi S3000H, Japan. The size of the HA micelles and losartan-HA micelles was analyzed using the DLS method, and the charge of the particles was analyzed on a Zetasizer instrument (Nano-Z590, Malvern Instruments, Worcestershire, UK).

### Conjugation of HA-Micelles with Fluorescent Dye

HA micelles were conjugated with Flamma^™^-552 and Flamma-774^™^ dye (BioActs, Incheon, Korea) using DCC/NHS chemistry to evaluate their targeting effects on human hepatic stellate cells (hHSC) and the biodistribution of HA micelles. The free carboxylic acid groups of HA were reacted with the amine-activated florescent probes in the presence of DCC and NHS. Briefly, HA micelles were dissolved in DMSO, and Flamma^™^-552 (Λ_abs_-551 nm, Λ_em_-570 nm) and Flamma^™^-774 (Λ_abs_-778 nm, Λ_em_-808 nm) were added at a fixed molar ratio of 1:100 (HA micelle: probe). Subsequently, DCC/NHS was added at a 5- and 10-fold molar ratio to the fluorescent probe present in the solution. The reaction mixture was stirred for one day in the dark and dialyzed using a membrane of MWCO = 3,500 to remove DMSO and unreacted materials. The lyophilized sample was analyzed for conjugation efficiency via an absorbance method using a multimode microplate reader (TECAN, Infinite M200 PRO, Männedorf, Switzerland).

### Cell Uptake Study of HA Micelles Labeled with Flamma^™^-552

Normal hepatocyte cells (FL83B) and hHSC were seeded at a density of 5 × 10^4^ cells in 8-well chamber slides (Lab-Tek2, New York, USA) and incubated in a humidified environment in CO_2_ at 37°C for one day. Stellate cell medium and F-12K medium containing 10 vol% FBS and 1 vol% pencillin-streptavidin-amphotericin B (Gibco Anti-Anti (100X),USA) were used for the hHSC and FL83B cells, respectively. After overnight incubation, HA micelles labeled with Flamma^™^-552 dye were added to each well at a concentration of 75 μg/ml. Following these treatments, the cells were incubated for another 2 hours. After the incubation period, the cells were washed with PBS and fixed with a 4% formaldehyde solution. The cells were then observed using a confocal microscope (Zeiss LSM 510, Oberkochen, Germany) equipped with HeNe (543 nm) and diode (405 nm) lasers for fluorescence at a magnification of 40x.

### Cell Viability Study

The cell viability of losartan-HA micelle-treated hHSC and FL83B cells was evaluated using the MTS assay. The cells were seeded into a 96-well plate at a density of 10^4^ cells/well. The cells were cultured in a CO_2_ incubator at 37°C in a humidified environment for one day. Losartan-HA micelles were added to the cells in triplicate to analyze their cytotoxicity over the concentration range of 0.001 μg/ml to 1,000 μg/ml. Triton was added at 5 μg/ml as a positive control. The cells were incubated for 24 hours after treatment. Then, 20 μl of MTS reagent was added to each of the treated wells and incubated for 4 hours. Finally, the absorbance at 490 nm was measured using a microplate reader.

### Immunocytochemistry Analysis

We divided the experimental samples into the 4 following groups: control, angiotensin, angiotensin plus losartan and angiotensin plus losartan-HA micelles. Angiotensin II can activate hHSC and induce overexpression of α-sma in hHSC. hHSC were seeded at a density of 5 × 10^4^ cells in 8-well chamber slides (Lab-Tek2,USA) and incubated in a humidified environment in CO_2_ at 37°C for one day. Stellate cell medium containing 10 vol% FBS and 1 vol% of pencillin-streptavidin-amphotericin B (Gibco Anti-Anti (100X), USA) were added to the cell culture. In the control group, only hHSC cells were added, and in the angiotensin group, angiotensin II was added to stimulate the hHSC cells. In the angiotensin plus losartan and the angiotensin plus losartan-HA micelle groups, free losartan and losartan-HA micelles (in PBS at 7.4 pH), respectively, were added 24 hours prior to the introduction of angiotensin II, which was added at a 1,000 nM concentration and incubated for another 2 hours. Anti-α-sma antibody (primary) and donkey anti-rabbit IgG (secondary) were used to evaluate the α-sma expression.

### Animal Experimental Procedure

C3H/HeN mice (5–6 weeks old, 20–25 g) were obtained from Jungang Lab Animal, Inc., Korea. The Chonnam National University Medical School Research Institutional Animal Care and Use Committee approved the experimental protocol (CNUHH 2014–148). Advanced hepatic fibrosis was induced in the C3H/HeN mice (n = 40) by injecting thioacetamide (TAA) dissolved in PBS, along with 10% ethanol in a water solution, by the intraperitoneal (IP) route three times per week. C3H/He mice were injected with PBS (7.4 pH) simultaneously by the IP route as a control (n = 3). The TAA concentration was incrementally varied (by 10 mg) from the starting concentration of 100 mg/kg based on the estimate weights of the mice. The combined TAA and ethanol administration continued for another 20 weeks.

After 20 weeks, the mice were divided into 3 groups of 12 mice each, as follows: HA micelle, losartan and losartan-HA micelle groups. Here, the HA micelle group can be considered to be the control group for this experiment. Losartan-HA micelles were administered 5 times, with a 3-day gap between each intravenous injection (300 μg/kg of losartan for 6.30 mg/kg of losartan-HA micelle), and a similar protocol was used for the HA micelle group without losartan loading. In the losartan group, free losartan was administered via oral gavage for the same period of time and frequency (300 μg/kg).

### Biodistribution of HA Micelles Labeled with Flamma^™^-774

HA micelles labeled with Flamma^™^-774 was injected into fibrotic (n = 3) and normal mice (n = 3) at 5 mg/kg concentration. For the *ex vivo* organ biodistribution study, the mice were sacrificed at 2-day time intervals, and their organs were harvested. Tissue biodistribution was analyzed using an IVIS Lumina (Xenogen, Toronto, USA) imaging system with indocyanine green excitation and emission filters at an exposure time of one second.

### Biochemical Analysis and Hydroxyproline Assay

The C3H/HeN mice with induced fibrosis were treated with free losartan, HA micelle, or losartan-HA micelle and were sacrificed after the end of treatment. Then, 800 μl to 1 ml of blood was removed by the cardiac puncture method (n = 12). The blood was immediately stored at 4°C to assist the clotting process. After the clotting process, the blood was centrifuged at 2500 rpm for 10 minutes to isolate plasma from the clotted blood. Blood plasma was analyzed for alanine amino transferase (ALT), aspartate amino transferase (AST), creatinine (CK) and lactate dehydrogenase (LDH) levels using Hitachi 7600 Automatic analyzer (HITACHI, Hitachi Koki Co., Ltd, Tokyo, Japan).

Hydroxyproline level was analyzed from losartan-HA micelle and PBS treated liver (n = 8), by acid hydrolysis process for hydroxyproline estimation. In brief, liver isolated from mice was weighed and 10 mg was hydrolyzed for 24 hours in a glass screw-thread vial with a teflon cap using 100μl distilled water and 100μl 10N HCL. After cooling, the hydrolysed sample was centrifuged at 10,000 rpm for 3 minutes to separate black residue from clear supernatant. 10μl of supernatant, 100μl of 1X chloramine T solution was added and incubated at room temperature for 20 minutes. 100μl of 1X DMAB was added next and incubated for 30 minutes. Finally, optical density was calculated at 530 nm using microplate reader.

### Histopathologic Study

Liver was excised from the mice after treatment with free losartan, HA micelle, and losartan-HA micelle and the liver tissue samples for the histopathological examination were washed with 0.9% NaCl solution and fixed in 10% formalin solution (n = 4). After standard tissue processing procedures, the tissues were embedded in paraffin. The 6 μm thick sections were prepared and stained with hematoxylin–eosin (HE) and masson Trichrome. One pathologist evaluated the fibrotic degree of liver based on METAVIR scoring system [24]. Paraffin embedded liver sections were used to conduct immunohistoflourescence study to evaluate α-sma expression. Briefly, free losartan, HA micelle, and losartan-HA micelle treated mice was sacrificed and liver was isolated and fixed in 4% PFA. After sectioning of paraffin embedded liver, anti-α-sma and donkey anti-rabbit IgG was used for the immunohistoflourescence study. For Immunohistochemistry SMA, Goat anti-rabbit IgG (HRP) as secondary antibody with DAB staining was used. All experiments were performed as per manufacture protocol.

### Statistical Analysis

Results are conveyed by mean ± standard error of the mean or mean ± standard deviation. Statistical analysis was established by Student unpaired *t* test and Fisher’s exact test using GraphPad QuickCalcs software. Differences were considered significant if *P*<0.05.

## Results

### Synthesis of Losartan-HA Micelle

**Fig 1:**
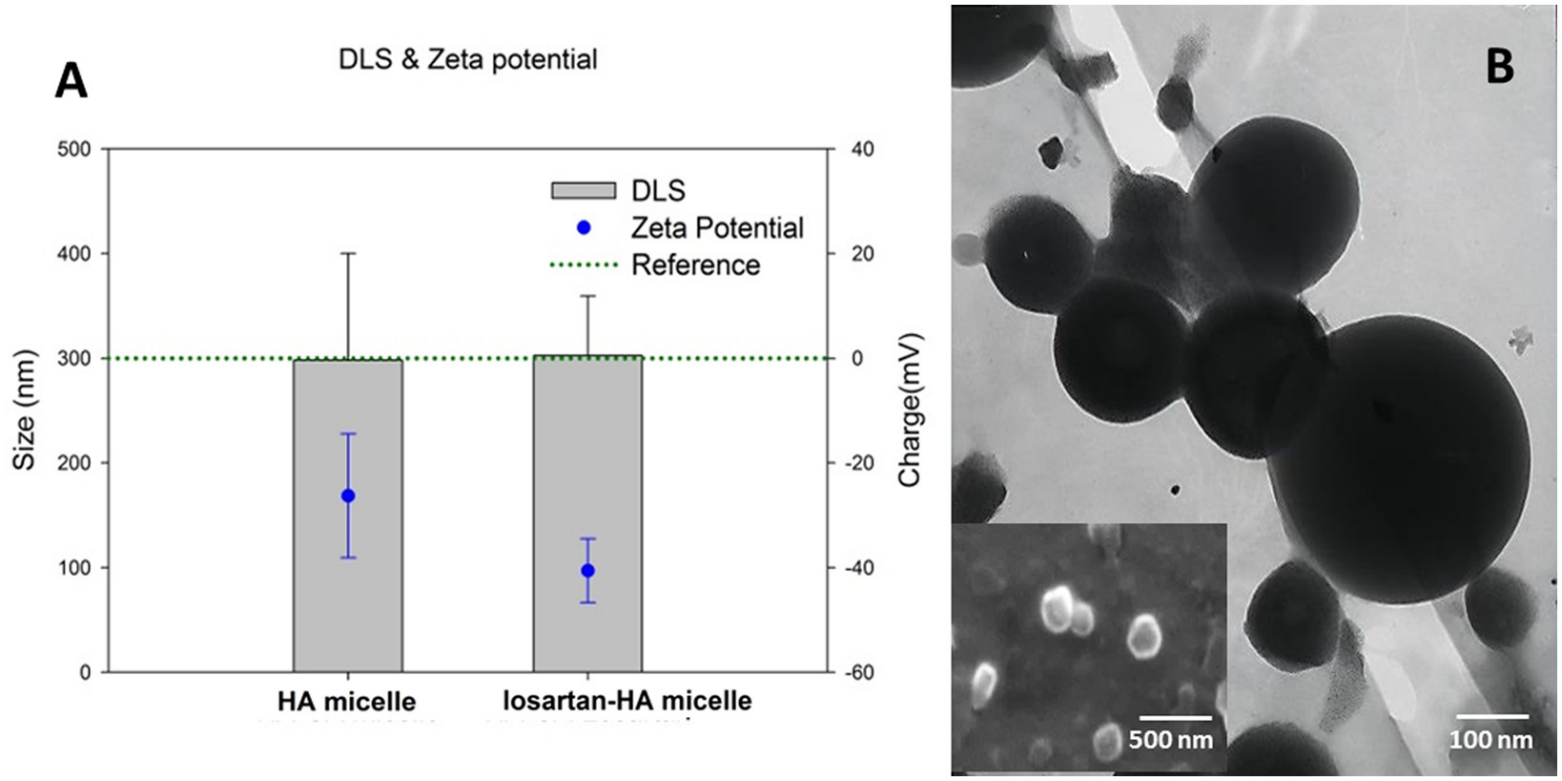
Physicochemical characteristics of HA micelle and losartan-HA micelle. **A.** Size measurement by DLS and surface charge measurement by zeta potential of HA micelle and losartan-HA micelle in PBS at 7.4 pH. HA micelle and losartan-HA micelle was taken at 100 μg/ml concentration and bath sonicated (Power Sonic410,Hwashin,Korea) for 5 minutes at room temperature and measured; **B.** TEM image of a SPION loaded HA micelle and SEM image of losartan-HA micelle (inset). The size of losartan-HA micelle was estimated to be around 300nm based on SEM and DLS result whereas the morphology of particle was confirmed from TEM and SEM image.

Losartan-HA micelle was synthesized by oil-in-emulsion method with 10:1 and 10:3 ratio of HA micelle to losartan. However, HPLC analyses revealed 10:3 has better drug loading efficiency compared to 10:1 even though its encapsulation efficiency was 83% (Table 1). Therefore we proceeded with 10:3 ratio for our further studies. DLS measurement revealed size of losartan-HA micelle to be 300 nm which was validated by SEM. Zeta potential of losartan HA micelle was –40 nm which explains good stability of micellar structure and solubility in water (Fig 1).

**Table 1:** Physicochemical characterization of losartan-loaded HA micelle. The data are presented as the mean ± SD.

Sample	Drug/Carrier(w/w)%	Size(nm)	Zeta Potential(mV)	CMC(μg/ml)	EE(%)	DL(%)
losartan-HA micelle	10%	--	--	--	83	8.3
	30%	300±25	-40±5	40±10	65	19.5

CMC: Critical micellar concentration, E.E: Encapsulation efficiency, DL: Drug loading

### Cell Uptake Study

**Fig 2:**
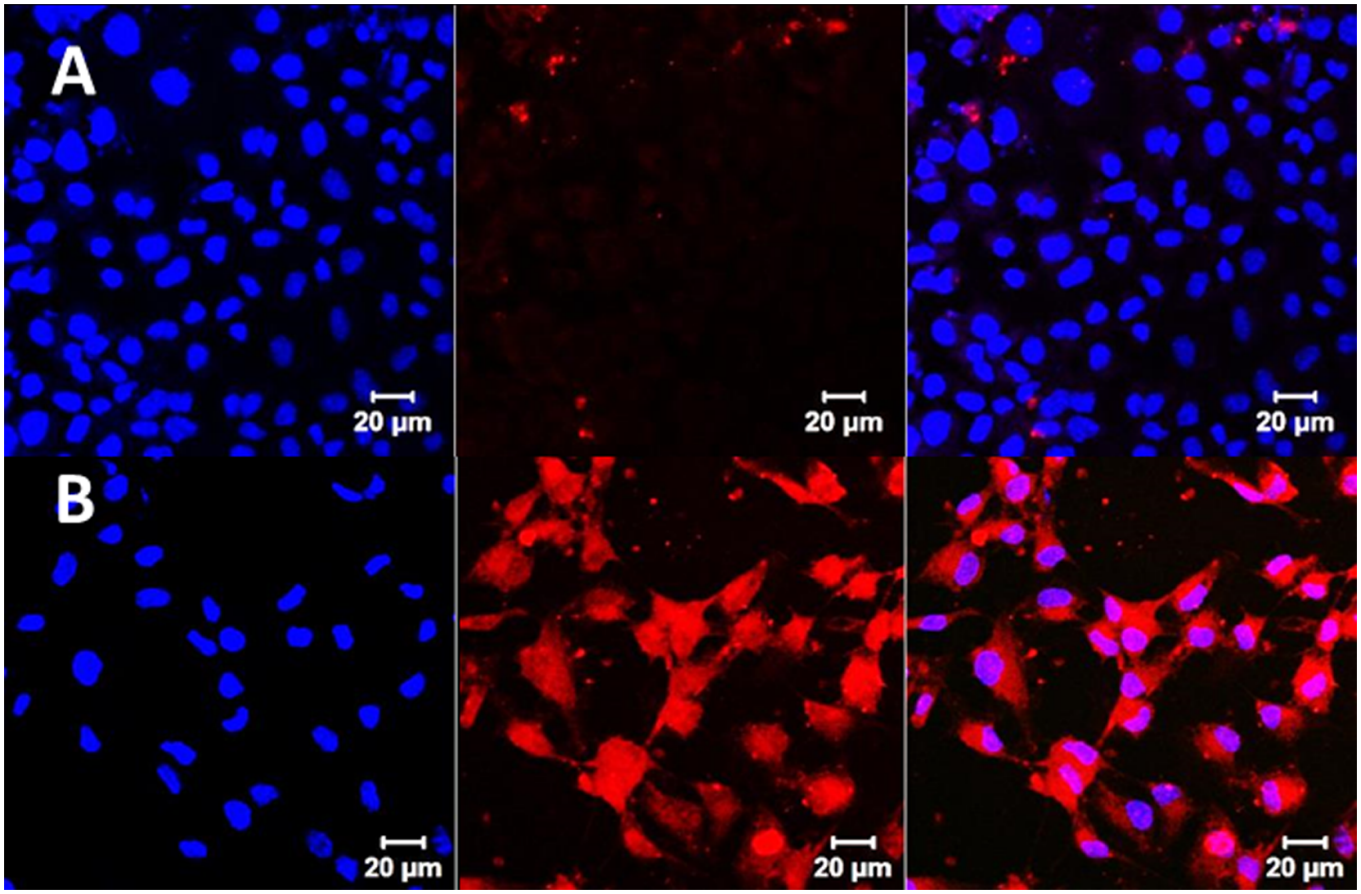
Cell uptake study of HA micelle labeled with Flamma^™^552 dye by confocal microscopy. **A.** Representative picture of FL83B cells incubated in 70 μg/ml of HA micelle labeled with Flamma^™^552. Accumulation of HA micelle labeled with Flamma^™^552 in FL83B cells was found to be minimal; **B.** Representative picture of hHSC cells incubated with 70 μg/ml of HA micelle labeled with Flamma^™^552. HA micelle labeled with Flamma^™^552 is clearly seen in HSC with more fluorescent intensity than FL83B. The incubation period was 2 hours for all experiments. Blue color represent uptake of DAPI stain in nucleus.

We compared the cell uptake efficiency of HA micelle labelled with Flamma^™^552 in FL83B cell line and hHSC cell line. FL83B cell line represent normal hepatocytes whereas hHSC cell line has the angiotensin 1 receptors which is the main target for losartan receptors. Confocal image revealed that hHSC showed higher uptake of HA-micelle than FL83B (Fig 2) based on stronger fluorescence intensity mediated by Flamma^™^552 dye.

### Cell Viability Study

**Fig 3:**
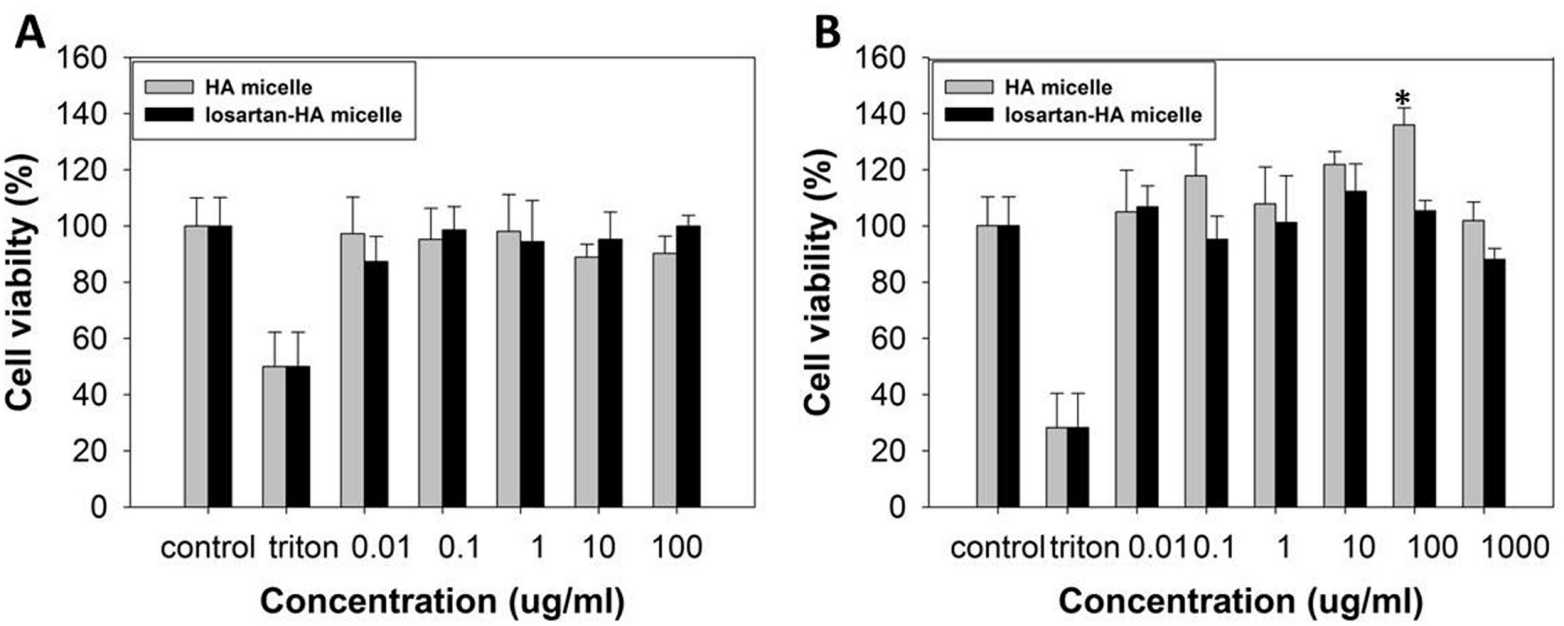
*In vitro* cytocompatibiliy of HA micelle and losartan-HA micelle (in PBS, pH 7.4). **A.** hHSC; **B.** FL83B cell line. MTS assay of HA micelle and losartan-HA micelle has minimum toxicity effect even at high concentration (100 and 1,000 μg/ml). MTS assay data show the mean cell viability of quadruplicate samples ± SD. Losartan-HA micelle at 100 μg/ml showed significantly higher cell viability (*P <0.01) relative to the control in FL83B cell line. All other treatment does not show significant change in cell viability compared to control in both hHSC and FL83B cell lines (P>0.05).

The micelle was evaluated of its toxicity in losartan-HA micelle. When treated in hHSC and FL83B cells, it was found to be non-toxic upto 1,000 μg/ml and 100 μg/ml respectively (Fig 3). Losartan-HA micelle doesn’t cause cell death to HSC s and FL83B cell lines at even higher concentration.

### Immunocytochemistry Evaluation

**Fig 4:**
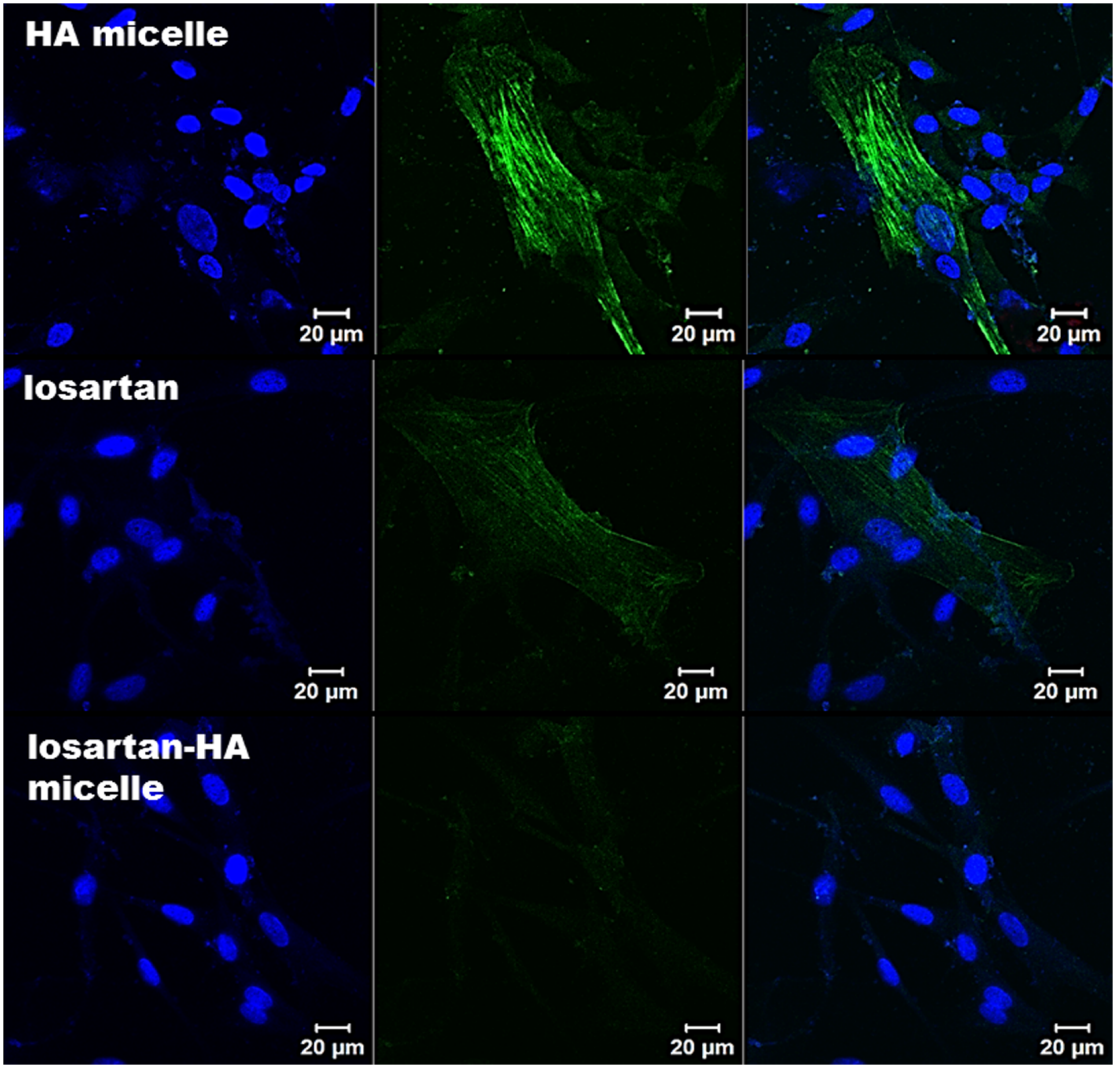
Confocal microscopy imaging of α-sma expression in hHSC cell. In HA micelle group, hHSC cells were incubated for 24 hours with HA micelle prior to 2 hour incubation with 1,000 nM of angiotensin 2. When angiotensin 2 is taken up by cells via angiotensin 1 receptor mechanism, expression of α-sma indicate the possible activation of HSC. In losartan group hHSC cells were incubated for 24 hours with 1,000nM losartan prior to 2 hour incubation with 1,000 nM of angiotensin 2. The angiotensin 1 receptors have been blocked by losartan which minimized expression of α-sma. In losartan-HA micelle group, hHSC cells were incubated for 24 hours with 1,000nM losartan-HA micelle prior to 2 hour incubation with 1,000 nM of angiotensin 2. Losartan-HA micelle helped in suppressing expression of α-sma more effectively. Blue color indicate DAPI stain.

Control group showed minimal expression of α-sma expression represented by green flourescence of secondary antibody. However angiotensin I group showed intense green fluorescence that indicates activation of hHSC. Both losartan and losartan-HA micelle groups 24 hours before addition of angiotensin showed marked decrease fluorescence. Especially losartan-HA micelle group did not show fluorescence due to better solubility in cell culture medium and cell uptake efficiency by losartan-HA micelle (Fig 4).

### Biodistribution of HA Micelle Labelled with Flamma^™^-774

**Fig 5:**
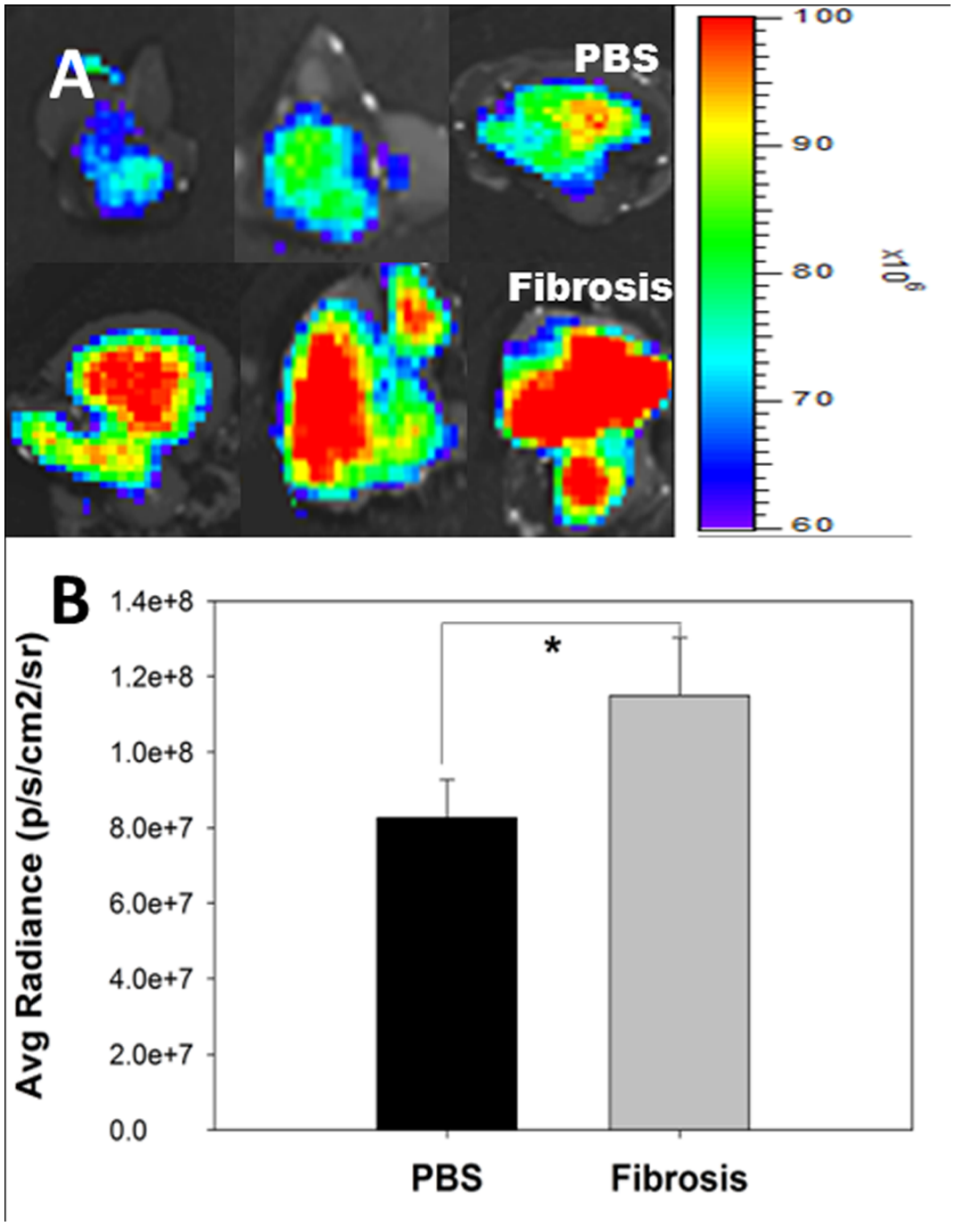
*Ex vivo* profile of HA micelle labelled with with Flamma^™^ 774 in the liver. (A) PBS treated normal mice shows accumulation of HA micelle labelled with with Flamma^™^ 774 (Top). TAA/Ethanol treated mice with liver fibrosis shows marked increased accumulation of HA micelle labelled with with Flamma^™^ 774 by CD44 receptor mediated uptake in HSC (Bottom). (B) Fluorescence intensity was quantified by the region-of-interest (ROI) method. The data are presented as the mean ± SEM. *P <0.05 relative to the liver ROI.

We analyzed the biodistribution of HA micelle labelled with Flamma^™^-774 in fibrotic and normal liver of mice (n = 3) (S2 Fig). After injecting 5mg/kg of HA micelle in both groups, ex-vivo image showed statistically significantly higher fluorescence intensity in fibrotic liver rather than normal liver (*P*<0.05). Due to fibrotic state of liver there is a chance of slower clearance of HA micelle labelled with Flamma^™^-774 in fibrotic liver compared to normal liver (Fig 5).

### Biochemical Estimation and Collagen Content in Liver

**Fig 6:**
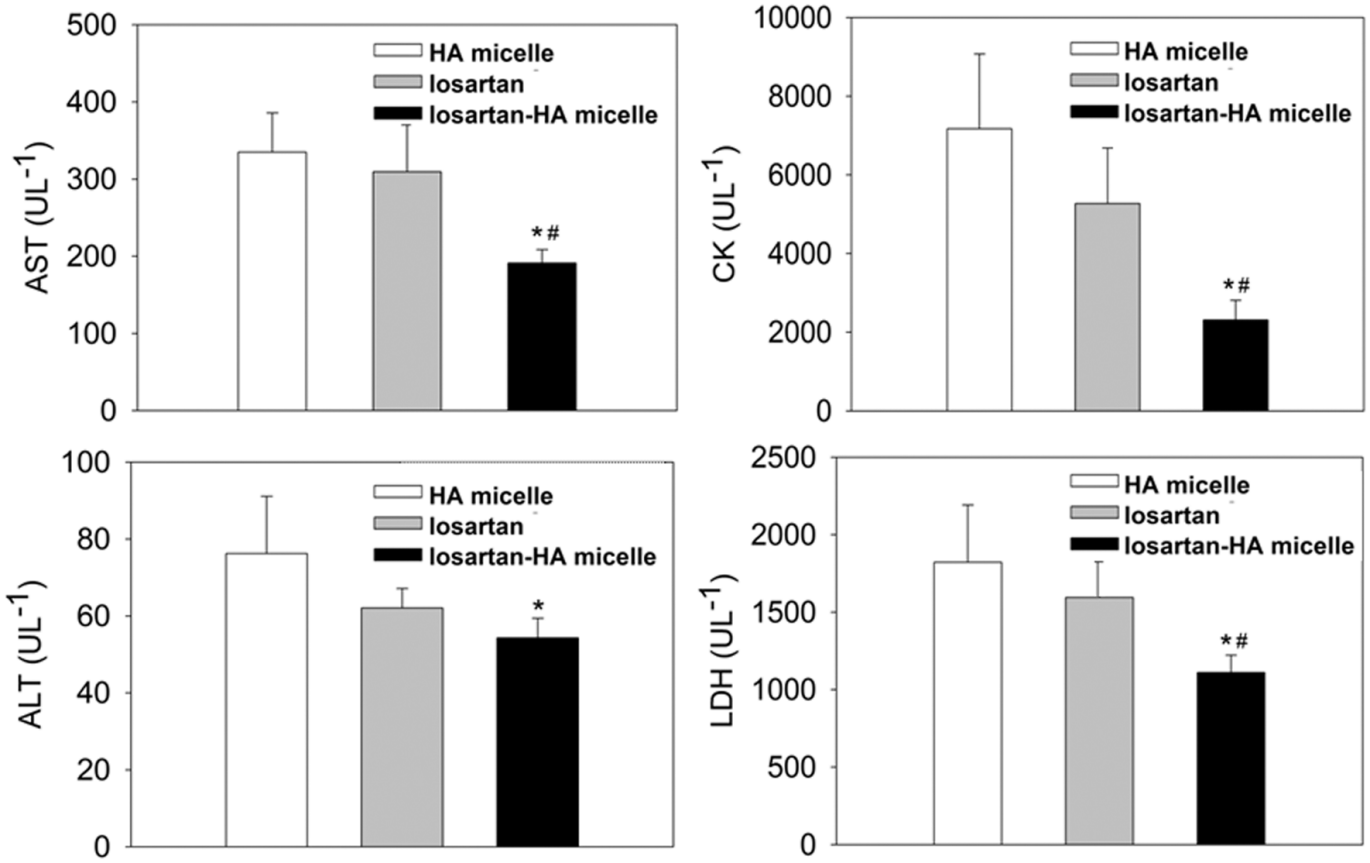
Blood biochemical estimation of AST, ALT, CK and LDH of HA micelle, losartan and losartan-HA micelle treated mice group after 20 weeks fibrosis induction by TAA/Ethanol treatment. Losartan-HA micelle group have statistically significant lower AST/ALT, CK and LDH values compared to HA micelle and losartan group which indicate better treatment effect due to successful losartan delivery to hepatic stellate cells in liver. The data are presented as the mean ± SEM. *P <0.05 and ^#^P <0.05 relative to HA micelle and losartan group, respectively.

From the blood serum collected from fibrosis induced C3H/HeN mice, biochemical analysis of losartan-HA micelle revealed significant (P ≤ .05) decrease in ALT, AST, CK and LDH level compared to other groups. HA micelle group showed high ALT, AST, CK and LDH values which can be attributed to the constant TAA/Ethanol administration for 20 weeks and ineffective treatment. Oral gavage treatment of free losartan gave lower serum values than HA micelle group showing minor response to losartan in free form. Losartan-HA micelle group showed better response in serum values (Table 2), indicating successful delivery to drug payload to the target site and angiotensin receptor blockade with better efficiency than free losartan or HA micelle group (Fig 6). AST/ALT ratio of all the groups are found to be having ratio more than 2:1 which indicates test to be highly specific and sensitive to liver damage. Although, losartan-HA micelle AST/ALT ratio is lower than HA micelle and losartan group which signifies good treatment response to losartan-HA micelle (Table 3).

**Table 2:** Blood biochemical estimation of AST, ALT, CK and LDH. The data are presented as the mean ± SEM with statistical significance and p-value less than 0.05 relative to HA micelle and losartan group, respectively.

Group	ALT (UL^-1^)	AST (UL^-1^)	CK (UL^-1^)	LDH (UL^-1^)
HA micelle	76.25 ± 14	334.83 ± 50	7174.44 ± 1898	1822.22 ± 368
losartan	62.09 ± 5	309.45 ± 62	5271.62 ± 1410	1594.75 ± 265
losartan-HA micelle	54.33 ± 5	191.08 ± 17	2309.00 ± 502	1111.11 ± 111

**Table 3:** AST/ALT ratio HA micelle,losartan and losartan-HA micelle group.

Group	AST/ALT
HA micelle	4.4
losartan	4.9
losartan-HA micelle	3.5

Hydroxyproline level in liver gives an indirect estimation of collagen that constitutes extracellular matrix. As fibrosis progress extra cellular matrix (ECM) content in liver increases which can be quantified by hydroxyproline assay. Losartan-HA micelle group showed marked decrease in hydroxyproline level compared to HA micelle group. This result showed that losartan-HA micelle can block the angiotensin 1 receptors on HSC and de activate them from releasing ECM content (S3 Fig).

### Histopathologic Study

**Fig 7:**
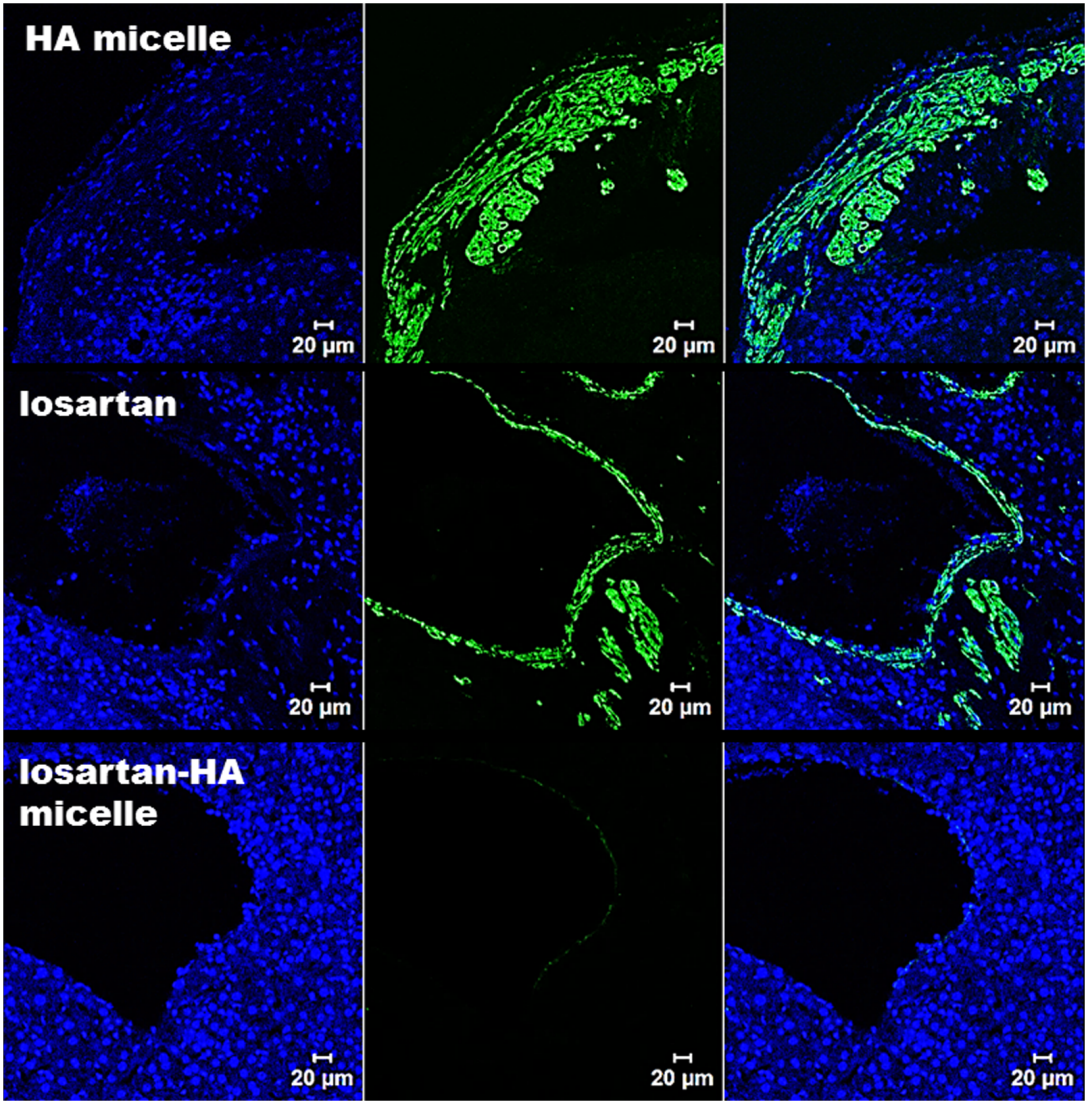
Confocal microscopy imaging of α-sma expression in liver section. In HA-micelle group, mice injected with HA micelle shows high expression of α-sma as the fibrotic state of liver have increased proliferation of activated HSC which express α-sma (represented by green fluorescence). In losartan group mice injected with losartan reveals similar expression profile of α-sma compared to HA micelle treated mice group as the oral losartan have failed to reach the target region (activated HSC). In losartan-HA micelle group, mice injected with losartan-HA micelle have almost no expression of α-sma which indicate successful delivery of losartan encapsulated losartan-HA micelle in the target region. Blue color indicate DAPI stain.

**Fig 8:**
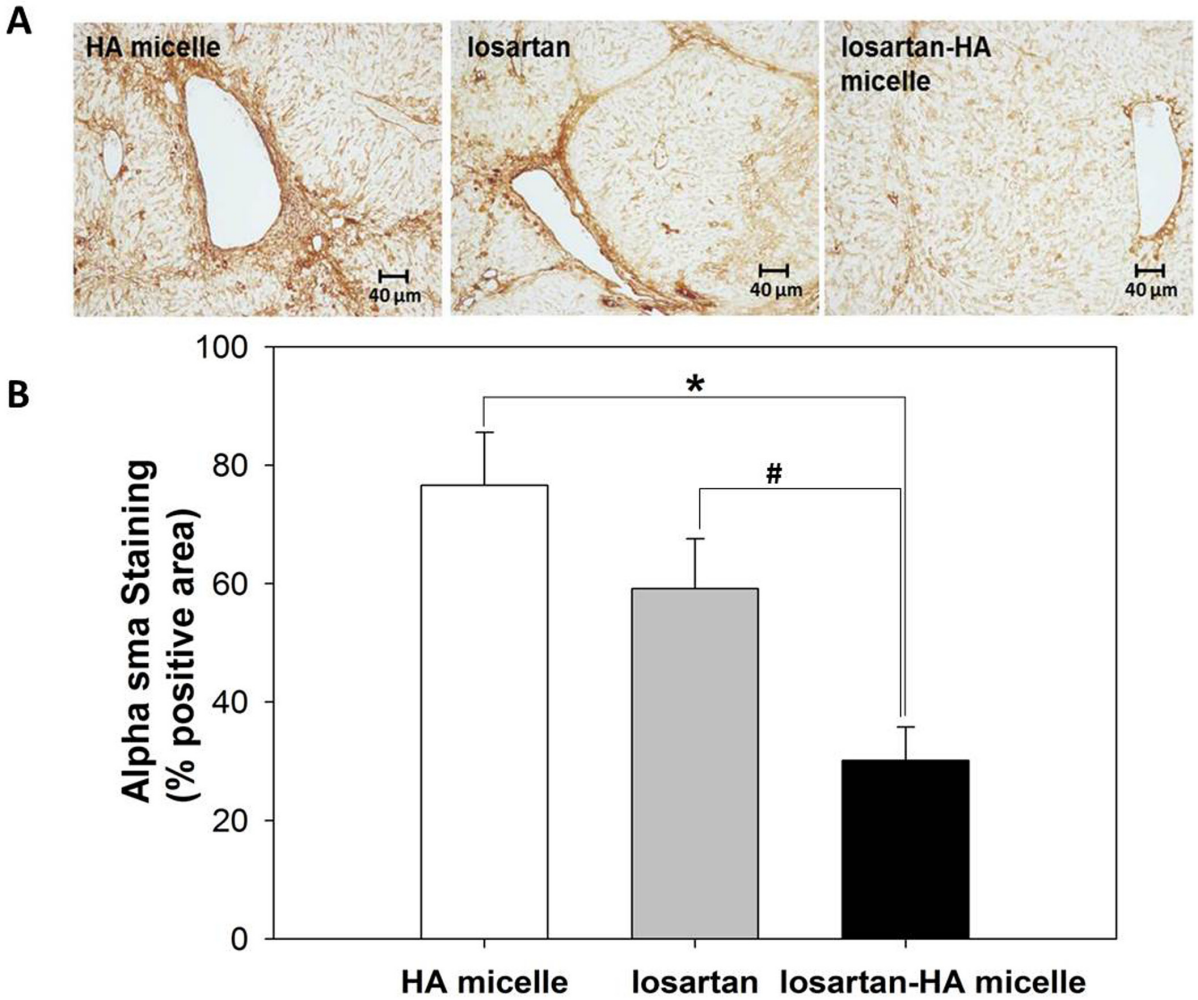
Immunohistochemical analysis of α-sma expression in liver section. (A) Representative images of smooth muscle actin immunohistochemical staining in liver. (B) The expression of α-sma in losartan-HA micelle group is significantly lower than those of the HA micelle and losartan groups. The proportion of areas immunopositive for smooth muscle actin was measured using IHC profiler plugin in ImageJ (NIH image program, version 1.49) software as mean percent positive areas. The data are presented as the mean ± SD *P <0.001 and ^#^P <0.001 relative to HA micelle and losartan group respectively.

In order to explore the effect of losartan-HA micelle in fibrotic liver α-sma activation was analyzed qualitatively. Activated HSC was represented by intense green fluorescence in immunofluorescence of HA micelle and losartan group. However losartan-HA micelle group exhibited no expression of α-sma (Fig 7). Quantitative assessment of α-sma activation was done using immunohistochemical staining in liver section The expression of α-sma in losartan group was found to be lower than HA micelle group. Whereas expression of α-sma in losartan-HA micelle group was estimated to be lower than both HA micelle and losartan group (P<.001), which proves the improved ability of losartan-HA micelles to deliver losartan to the HSC cells compared to the other groups (Fig 8).

The HA micelle- and losartan-treated groups revealed bridging fibrosis that indicate advanced hepatic fibrosis (S4 Fig). The losartan-HA micelle group demonstrated septal fibrosis but no bridging fibrosis. This result indicates that losartan-HA micelle treatment resulted in improved attenuation of liver fibrosis in C3H/HeN mice (S5 Fig).

## Discussion

In our study, we have developed a HSC-targeting HA micellar system loaded with losartan to prevent the progression of fibrosis. Amphiphilic conjugates of HA can form hydrophobic drug carriers in aqueous conditions. HA micelles can carry hydrophobic drugs such as losartan to the liver without affecting the payload. Recently, nanoparticle-based systems have been evaluated as treatments for hepatic fibrosis [25, 26]. A plant-based polyphenol similar to curcumin was modified into a polymeric nanoparticle to improve the drug’s solubility profile and increase bioavailability, and this formulation has been demonstrated to be effective against CCl_4_-induced liver cirrhosis by enhancing the levels of antioxidants in the liver. Silymarin, another experimental antifibrotic agent, was coated on gold nanoparticles, tested as a therapy against CCl_4_-induced liver cirrhosis in mice and found to exhibit minimal side effects. Compared with previous studies [25, 26], our work is unique in the use of an HA-based nanomicellar system that actively targets the HSC for the delivery of losartan.

Our cell uptake studies demonstrated the preferential uptake of HA micelles labeled with Flamma^™^552 by hHSC cells compared with FL83B cells. Similar results were reported in a separate study in which hyaluronic acid (HA)-QDot conjugate accumulation was observed to be higher in HSCs than in the FL83B cell line [21]. Our result demonstrated that hHSC exhibits improved uptake compared to the FL83B cell line, which indicates an abundance of HA receptors (CD44 or RHAMM) that facilitate the effective accumulation of losartan-loaded HA micelles in the space of Disse in the liver, where the HSCs are located.

Losartan-HA micelles did not cause significant HSC and FL83B cell death, even at higher concentration. Although the targeted approach of losartan-HA micelle ensures escape from the general liver parenchyma in *in vivo* conditions, some possibility of losartan exposure to normal cells still remains. The nontoxicity of the micelles is important to prevent apoptosis of HSC cells and hepatocytes during antifibrotic therapy. HA is a glycosaminoglycan abundantly found throughout connective, epithelial, and neural tissues [15]. The polymer is clinically used in to treat osteoarthritis of the knee as an injectable material and in the synthesis of biological scaffolds for wound healing [16]. HA-based micelle systems can be utilized in clinical trial to treat hepatic fibrosis.

We developed an advanced liver fibrosis model using C3H/HeN mice with an improved fibrosis induction method, in which TAA injection and ethanol feeding was carried out simultaneously [27]. Though this method was successful in increasing the rate of liver fibrosis induction compared to TAA administration alone, the mortality rate soared from 18% to 40%. Therefore, a modification in the fibrosis induction treatment was adopted, wherein the TAA administration dosage was varied depending on the weight changes experienced by the animals (S1 Fig). This weight-adapted model of fibrosis has been previously studied in rats, which achieved 0% mortality [28]. The application of this TAA/ethanol-treated C3H/HeN mouse strategy proved to be effective. Our study demonstrated 0% mortality in mice and the successful induction of hepatic fibrosis. Previous works have been performed using a CCl_4_-induced mouse model of fibrosis. The TAA-induced fibrosis in our study has been reported to be the best model for studying alcohol-induced liver fibrosis [29]. The experimental results of our study can be more readily related to actual fibrosis regression studies conducted in humans.

Our study demonstrated, through anti-α-sma antibody immunostaining of hHSC cells and fibrotic liver, a marked reduction in smooth muscle actin in the losartan-HA micelle-treated group, which indicates the possible deactivation of the HSC. The percentage of α-sma was quantified by immunohistochemistry, which also showed significantly less accumulation of α-sma positive cells in losartan-HA micelle group. The effect of angiotensin II on the AT1 receptor on HSCs has been widely studied previously and has been found to serve as a good *in vitro* experimental indicator with which to study antifibrogenic responses [30]. The losartan group also exhibited mild suppression of α-sma, which could have resulted from the direct addition of losartan potassium into the medium compared to *in vivo* administration conditions in which the drug must cross the gastrointestinal barrier to reach the liver. HSC deactivation can be more effectively achieved by the losartan-HA micelle system.

HA micelles labeled with Flamma^™^774 demonstrated effective accumulation in fibrotic livers compared to normal livers, in the biodistribution study, with slower clearance of HA micelles labeled with Flamma^™^-774 in the fibrotic livers than in the normal livers. This result indicates that the overexpression of CD44 receptors on activated hHSC mediated the endocytosis of the HA micelles compared to the FL83B cell line [19]. Losartan-HA micelle uptake can be targeted to hHSC, thus increasing the drug delivery to the target site. CD44-based receptor-mediated uptake and the accumulation of HA micelles occurred at a higher rate in fibrotic livers because the proliferation of HSC is increased by over 10- to 20-fold [21].

Blood serum analysis provides a biochemical estimation of enzymes that directly or indirectly indicate collagen levels in the liver. Blood serum analysis also revealed significant decreases in the ALT, AST, CK and LDH levels in the losartan-HA micelle group compared with the other groups. This result is related to the improved liver conditions for activating fibrosis progression through HSC angiotensin 1 receptor blockade. The liver histology results indicated a decrease in bridging fibrosis and the clearance of macronodules in the losartan-HA micelle group.

Injecting losartan-HA micelle could affect fibrotic process in liver through change in perfusion and ROS generation. In addition, the effects of any agent which is supposed to be infused should take into consideration the whole organism [31]. Moreover, losartan can change hemodynamics. However, we did not performed perfusion and hemodynamic study after injection of losartan-HA micelle. This point is a limitation of our study. We think that further research is needed for evaluation of perfusion and hemodynamic changes after injection of losartan-loaded nanoparticle.

## Conclusions

Losartan-HA micelles demonstrated significant attenuation of hepatic fibrosis by a HSC-targeting mechanism in both in vitro and in vivo studies. Losartan-HA micelles are an attractive option for antifibrotic therapy that can prevent or control further liver injury that would otherwise lead to liver cirrhosis or HCC. These nanoparticles may also be useful as a theranostic agent to actively monitor HA micelle accumulation in the liver via a conjugated optical contrast agent.

## Supporting Information

S1 Fig Hepatic fibrosis induction treatment in C3h/HeN mice (A1, A2, A3). PBS treated mice and (B1, B2, B3) TAA/Ethanol treated mice. Red dotted line indicate the abdomen region size of mice with more cm^2^ area representing higher body weight due to better nourishment as compared to mal-nourished TAA treated mice.
(TIF)

S2 Fig *Ex vivo* biodistribution profile of HA micelle labelled with with Flamma^™^ 774 in the liver, skin, kidney, heart, lung, gut and spleen (n = 3) of (A) Fibrosis induced mice and (B) Normal mice. (TIF)

S3 Fig Hydroxyproline level of HA micelle, losartan and losartan-HA micelle with fibrotic mice liver after 20 weeks treatment with TAA/Ethanol (n = 8). Hepatic hydroxyproline level in losartan-HA micelle markedly decreased compared to oral losartan and HA micelle treated group. The data are presented as the mean ± SEM. *P <0.001 relative to HA micelle group.
(TIF)

S4 Fig Masson trichrome and H&E staining of mice liver tissue. (A&B) HA micelle group show bridging fibrosis that indicate advanced hepatic fibrosis. (C&D) Losartan group have fibrous bands (arrow) and irregular cellular parenchyma. (E&F) Losartan-HA micelle group show septal fibrosis. Liver tissue displaying fibrous bands is represented by green arrow head (B), central hepatic venules (CHV), parenchymal nodules (N). All images are taken at 40x magnification.
(TIF)

S5 Fig Pie chart evaluating fibrosis resolution in HA micelle, losartan and losartan-HA micelle groups by METAVIR scoring system. Statistical analysis was done using Fisher's exact test with p-value = 0.13. However there was a large difference between percentage of mice with cirrhosis (75% in HA micelle and losartan group vs 37.5% in losartan-HA micelle), which is biologically significant though not statistically significant.
(TIF)

S1 Schema Experimental Design for *in vivo* liver fibrosis regression study. (TIF)
