# Supplementary figures and images for: Effectiveness of Losartan-Loaded Hyaluronic Acid (HA) Micelles for the Reduction of Advanced Hepatic Fibrosis in C3H/HeN Mice Model
Source: PLoS One. 2015 Dec 29;10(12):e0145512. doi: 10.1371/journal.pone.0145512 (PMC4699854; doi:10.1371/journal.pone.0145512)

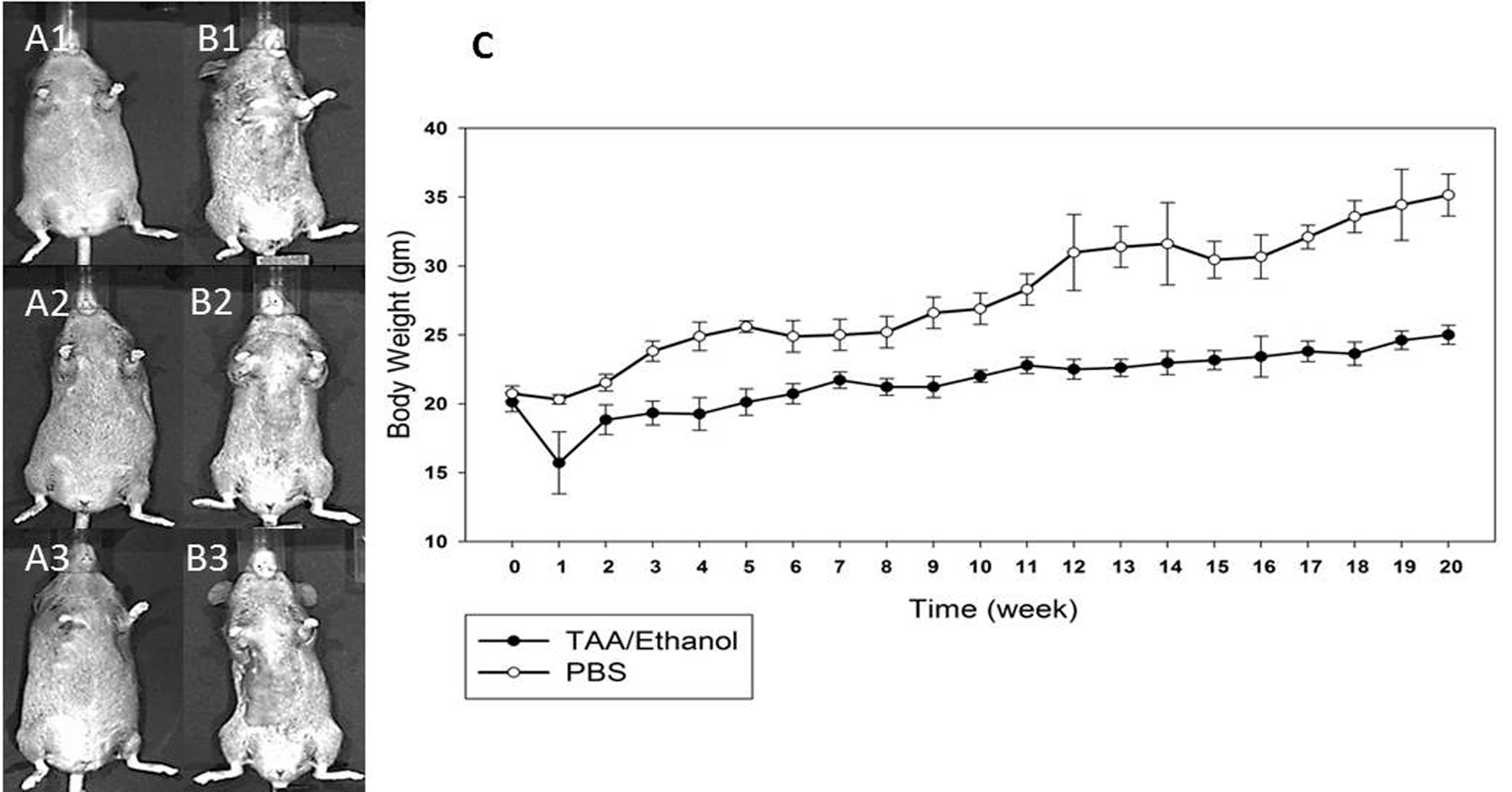

Supplement: S1 Fig — PBS treated mice and (B1, B2, B3) TAA/Ethanol treated mice. Red dotted line indicate the abdomen region size of mice with more cm2 area representing higher body weight due to better nourishment as compared to mal-nourished TAA treated mice. (TIF) [file pone.0145512.s001.tif]

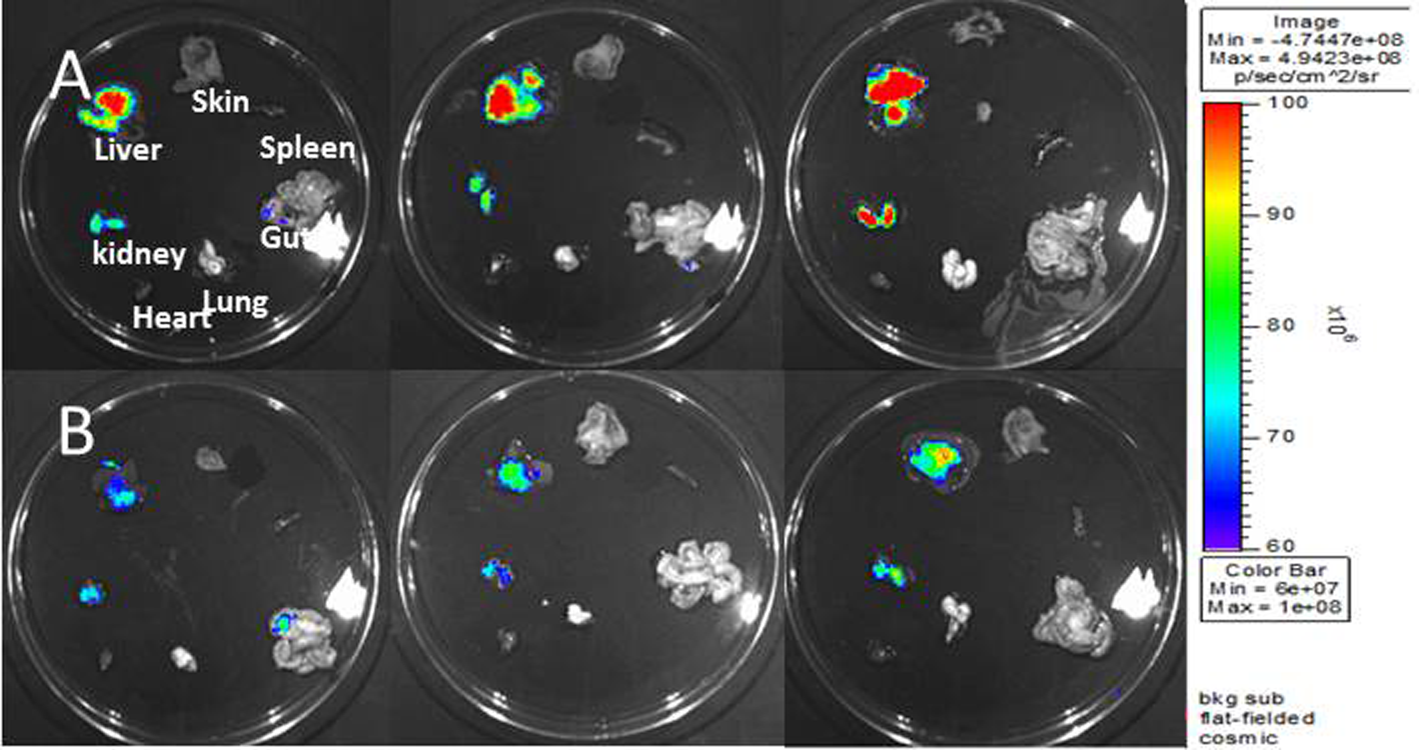

Supplement: S2 Fig — (TIF) [file pone.0145512.s002.tif]

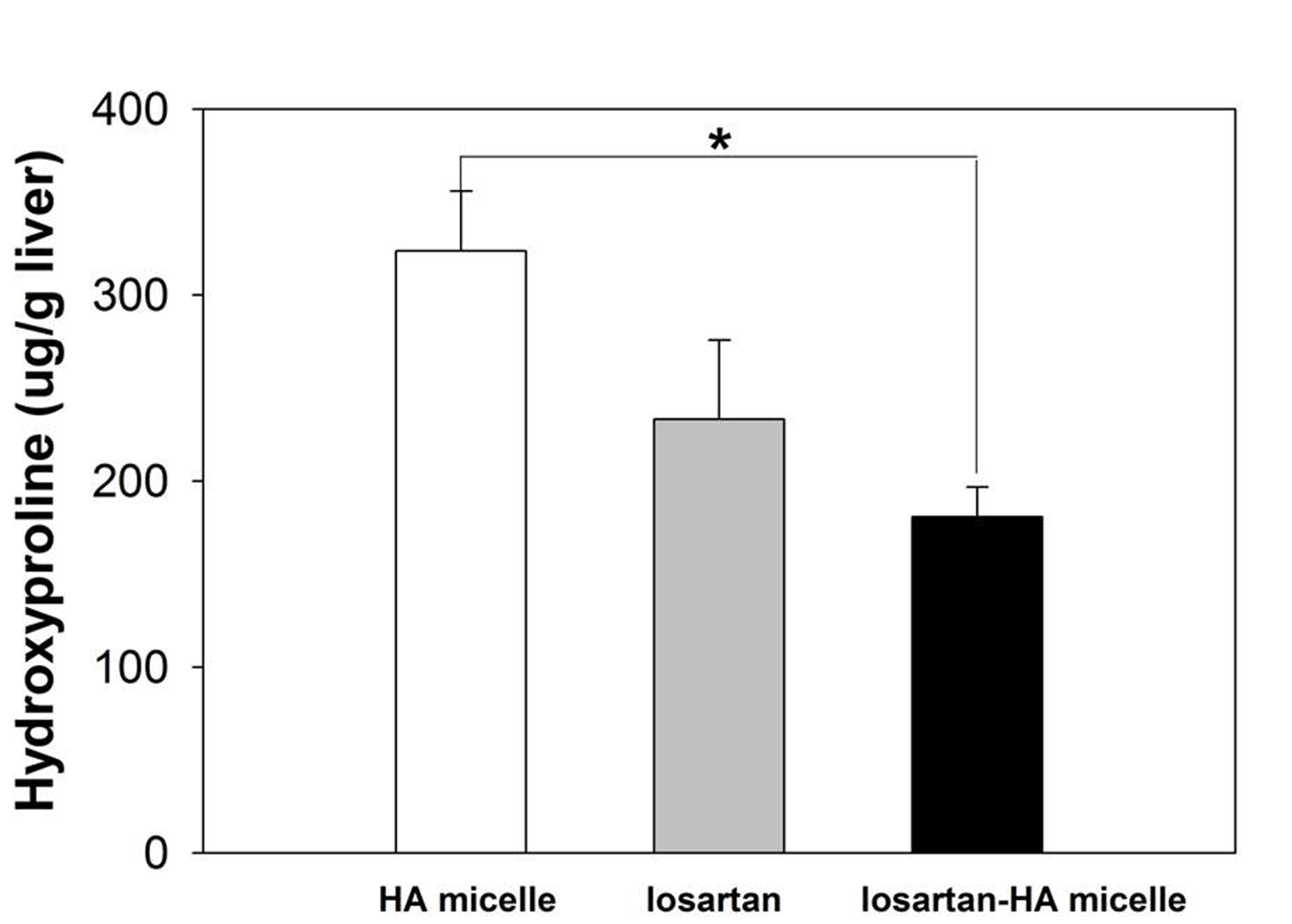

Supplement: S3 Fig — Hepatic hydroxyproline level in losartan-HA micelle markedly decreased compared to oral losartan and HA micelle treated group. The data are presented as the mean ± SEM. *P <0.001 relative to HA micelle group. (TIF) [file pone.0145512.s003.tif]

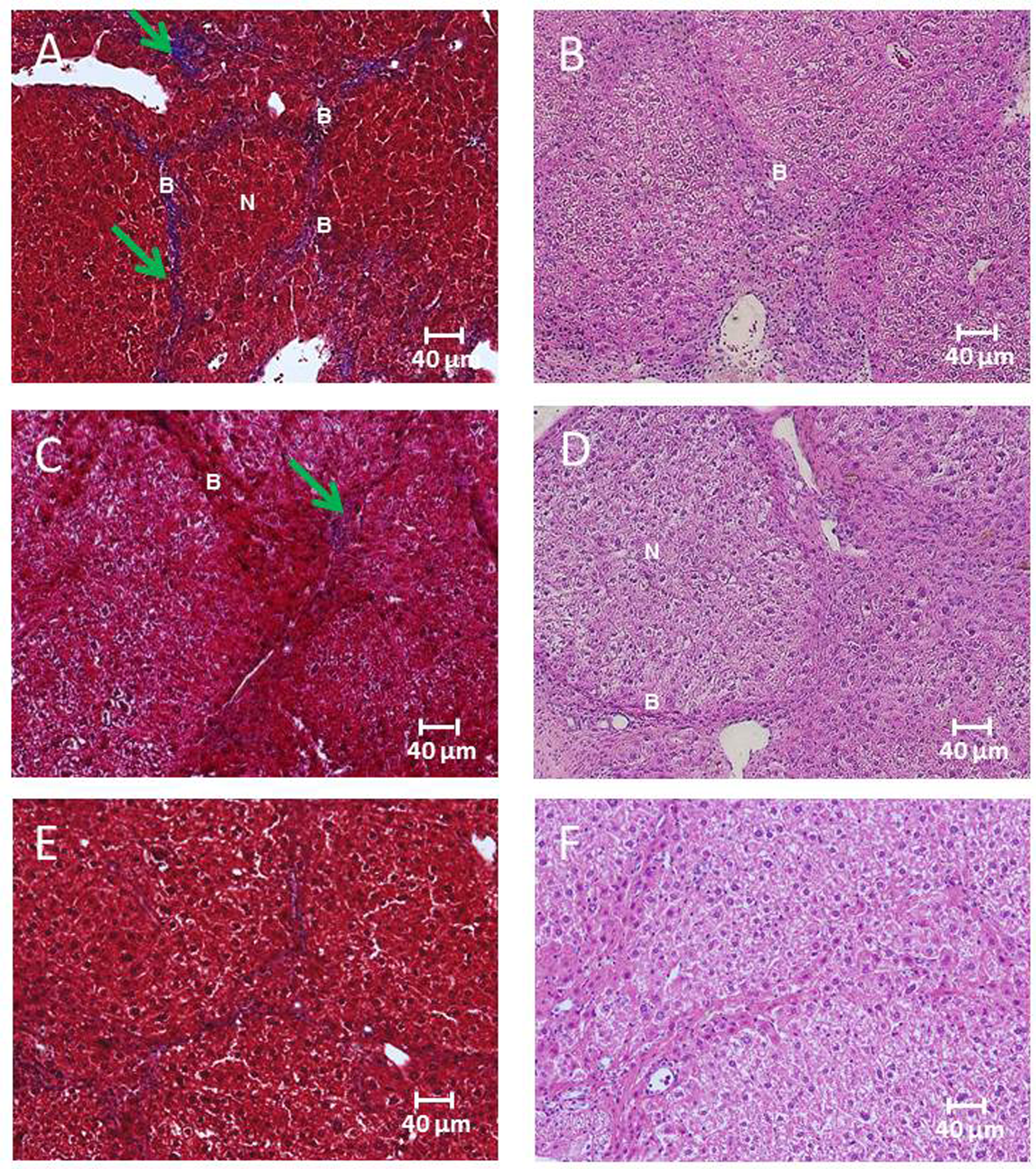

Supplement: S4 Fig — (A&B) HA micelle group show bridging fibrosis that indicate advanced hepatic fibrosis. (C&D) Losartan group have fibrous bands (arrow) and irregular cellular parenchyma. (E&F) Losartan-HA micelle group show septal fibrosis. Liver tissue displaying fibrous bands is represented by green arrow head (B), central hepatic venules (CHV), parenchymal nodules (N). All images are taken at 40x magnification. (TIF) [file pone.0145512.s004.tif]

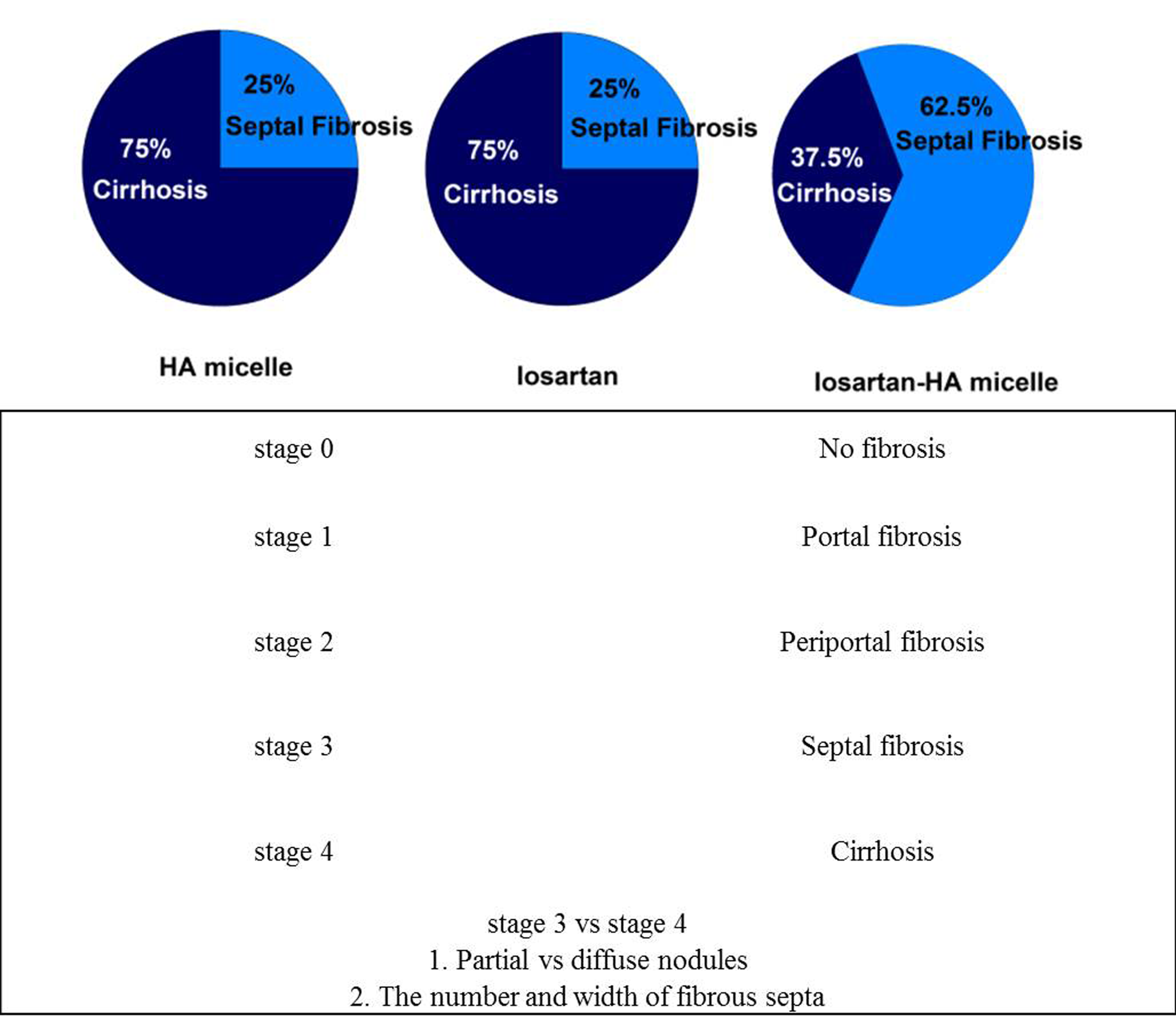

Supplement: S5 Fig — Statistical analysis was done using Fisher's exact test with p-value = 0.13. However there was a large difference between percentage of mice with cirrhosis (75% in HA micelle and losartan group vs 37.5% in losartan-HA micelle), which is biologically significant though not statistically significant. (TIF) [file pone.0145512.s005.tif]

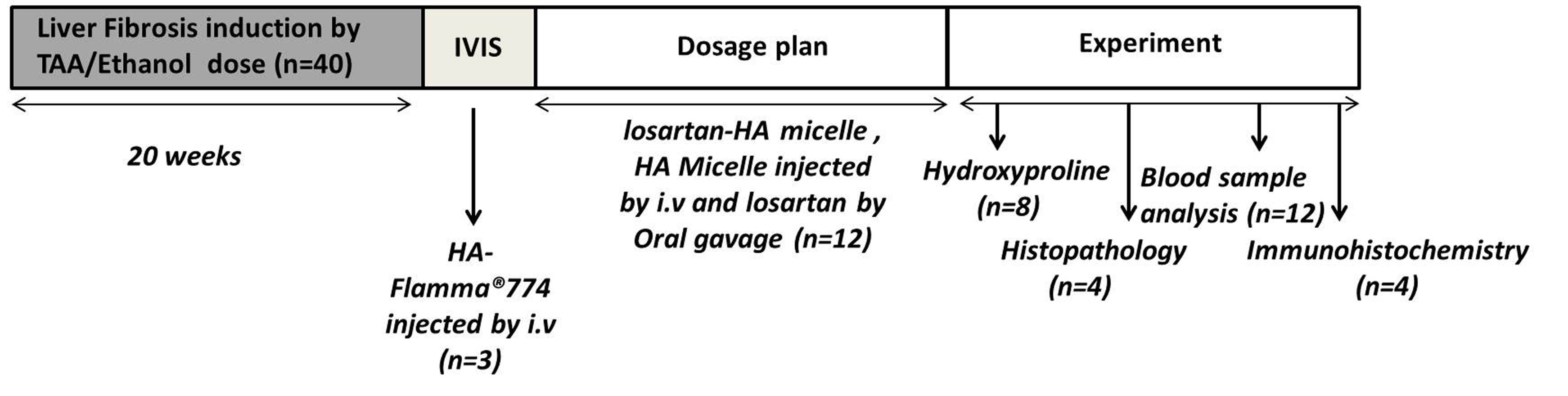

Supplement: S1 Schema — (TIF) [file pone.0145512.s006.tif]
